# RA-PR058, a novel ramalin derivative, reduces BACE1 expression and phosphorylation of tau in Alzheimer’s disease mouse models

**DOI:** 10.1080/19768354.2025.2459649

**Published:** 2025-02-07

**Authors:** Yongeun Cho, Jeongmi Lee, Jun-Sik Kim, Yeji Jeon, Sukmin Han, Heewon Cho, Yeongyeong Lee, Tai Kyoung Kim, Ju-Mi Hong, Yujeong Lee, Yujung Byun, Minshik Chae, Sunyoung Park, Leon F. Palomera, Sang Yoon Park, Hyunwook Kim, Soyeong Kim, Seongeun Kang, Jun-Goo Jee, Hongchan An, Joung Han Yim, Sung Hyun Kim, Dong-Gyu Jo

**Affiliations:** a School of Pharmacy, Sungkyunkwan University, Suwon, Republic of Korea; b Department of Neuroscience, Graduate School, Kyung Hee University, Seoul, Republic of Korea; c Division of Polar Life Sciences, Korea Polar Research Institute, Incheon, Republic of Korea; d Bio Research Dept., Ahngook Pharmaceutical, Gwacheon, Republic of Korea; e New Drug Development Center, Daegu-Gyeongbuk Medical Innovation Foundation (DGMIF), Daegu, Republic of Korea; f College of Pharmacy, Kyungpook National University, Daegu, Republic of Korea; g College of Pharmacy and Institute of Pharmaceutical Sciences, CHA University, Pocheon, Republic of Korea; h Department of Physiology, School of Medicine, Kyung Hee University, Seoul, Republic of Korea; i Samsung Advanced Institute for Health Sciences and Technology, Sungkyunkwan University, Seoul, Republic of Korea; j Biomedical Institute for Convergence, Sungkyunkwan University, Suwon, Republic of Korea; k Institute of Quantum Biophysics, Sungkyunkwan University, Suwon, Republic of Korea

**Keywords:** Alzheimer’s disease, BACE1, Tau, RA-PR058

## Abstract

Alzheimer’s disease (AD) is a multifactorial neurodegenerative disorder characterized by cognitive decline, anxiety-like behavior, β-amyloid (Aβ) accumulation, and tau hyperphosphorylation. BACE1, the enzyme critical for Aβ production, has been a major therapeutic target; however, direct BACE1 inhibition has been associated with adverse side effects. This study investigates the therapeutic potential of RA-PR058, a novel ramalin derivative, as a multi-targeted modulator of AD-related pathologies. The effects of RA-PR058 were evaluated *in vitro* and *in vivo*. *In vitro* studies used SH-SY5Y cells under oxidative stress conditions to assess BACE1 expression, while *in vivo* effects were studied in 3xTg-AD mice following one month of oral RA-PR058 treatment. Behavioral assessments, biochemical analyses, transcriptomic profiling, and pharmacokinetic evaluations were performed to determine the efficacy of RA-PR058. RA-PR058 significantly reduced oxidative stress-induced BACE1 expression *in vitro* and decreased cortical BACE1 expression in 3xTg-AD mice. *In vivo* treatment alleviated anxiety-like behavior and reduced tau phosphorylation at disease-relevant sites (Ser202/Thr205, Thr231, and Ser396). Transcriptomic analysis revealed RA-PR058-mediated gene expression changes related to central nervous system development, response to hypoxia, and neuroactive ligand–receptor interactions, suggesting broader regulatory effects on AD-related pathways. Pharmacokinetic analysis demonstrated that RA-PR058 exhibits high metabolic stability, minimal cytochrome P450 interactions, and moderate blood–brain barrier penetration. RA-PR058 demonstrates potential as a multi-target AD therapeutic by reducing BACE1 expression, tau hyperphosphorylation, and anxiety-like behavior, coupled with favorable pharmacokinetics. Additional studies are needed to assess cognitive effects and clarify molecular mechanisms, but RA-PR058 may represent a promising advancement in addressing AD’s complex pathology.

## 1. Introduction

Alzheimer’s disease (AD) is the most prevalent neurodegenerative disorder worldwide, characterized by progressive cognitive decline and memory loss. The global number of people affected by dementia is projected to rise from 57 million in 2019 to 150 million by 2050 (Author 2022). Despite extensive efforts to develop effective treatments, no curative therapy for AD currently exists.

The pathological hallmarks of AD include the excessive accumulation of β-amyloid (Aβ), neurofibrillary tangles comprised of hyperphosphorylated tau, neuroinflammation, synaptic and neuronal loss (Knopman et al. 2021), (Lee et al. 2024). Aβ is primarily produced in neurons through the amyloidogenic processing pathway of amyloid beta precursor protein (APP) (Cho et al. 2022). This pathway involves the sequential cleavage of APP by β-secretase and γ-secretase complex, which leads to the release of Aβ monomers (Cheng et al. 2015). These monomers, particularly Aβ_1-42_, aggregate into toxic oligomers, which lead to impairment of synaptic functions and contribute to cognitive decline observed in AD (Holtzman et al. 2011).

β-site amyloid precursor protein cleaving enzyme 1 (BACE1), a transmembrane aspartyl protease, is the β-secretase involved in the Aβ production (Luo et al. 2001). Since the cleavage of APP by BACE1 is the rate-determining step in the Aβ generation, BACE1 has been regarded as a primary therapeutic target for AD treatment (Woo et al. 2011; Vassar et al. 1999). However, despite extensive efforts to develop BACE1 inhibitors, many candidates have failed in clinical trials. These failures may be attributed to off-target effects of BACE1 inhibition, leading to the unintended suppression of physiological substrates critical for normal function (Ou-Yang et al. 2018; Willem et al. 2006). Thus, modulating BACE1 activity or expression, rather than complete inhibition, may represent a more viable therapeutic approach (Bahn and Jo 2019; Bahn et al. 2019).

Neurofibrillary tangles are another pathological feature of AD, composed of abnormally hyperphosphorylated tau proteins (Querfurth and LaFerla 2010). Tau is a microtubule-associated protein that plays a critical role in microtubule stabilization. Physiologically normal tau protein is involved in the assembly of microtubules, however, aberrantly hyperphosphorylated tau lacks affinity for microtubules and makes helical filament structures which are insoluble (Weingarten et al. 1975). These aggregates of abnormal tau proteins are neurotoxic like oligomeric Aβ and deteriorate cognition (Khlistunova et al. 2006; Santacruz et al. 2005).

Ramalin (γ-glutamyl-N′-(2-hydroxyphenyl)hydrazide) is a natural compound derived from the Antarctic lichen *Ramalina terebrata*, and previous studies have demonstrated its antioxidant, antibacterial and anticancer properties (Suh et al. 2017; Paudel et al. 2011; Paudel et al. 2010). Additionally, ramalin has been reported to exert anti-inflammatory effects, including inhibition of nitric oxide (NO) production in response to lipopolysaccharide (LPS) stimulation (Kim et al. 2021).

To enhance the therapeutic potential of ramalin for AD, we designed and synthesized derivatives of ramalin with various structural groups. Among these derivatives, RA-PR058 demonstrated high metabolic stability and moderate blood–brain barrier penetration. In this study, we assessed the therapeutic effects of RA-PR058 on AD-related pathologies using both *in vitro* and *in vivo* AD models. Furthermore, RNA-sequencing analysis of cortices from 3xTg-AD mice treated with RA-PR058 was conducted to examine transcriptomic changes. Our findings suggest that RA-PR058 is a promising novel therapeutic for the treatment of AD.

## 2. Materials and methods

### 2.1. Animals

3xTg-AD (B6;129-Tg(APPSwe,tauP301L)1Lfa *Psen1^tm1Mpm^*/Mmjax) mice and their littermate wild-type (WT) mice were used in this study. Each experimental group consisted of 4–8 mice, with groups designated as WT, 3xTg-AD Vehicle, and 3xTg-AD RA-PR058. All mice were maintained in a 12-hour light/dark cycle with free access to food and water. 7-month-old age of mice were administered with 20 mg/kg of RA-PR058 orally once a day for 1 month followed by the elevated plus maze test. All animal experimental procedures were approved by the Institutional Animal Care and Use Committee of Sungkyunkwan University (SKKUIACUC2022-10-41-1).

### 2.2. Synthesis of RA-PR058

Synthesis of *tert*-butyl (*S*)-(1,1-difluoro-5-(2-(2-fluorophenyl)hydrazineyl)-5-oxopentan-2-yl)carbamate (RA-PR058-Boc): A 100 mL round flask equipped with a magnetic stir bar was loaded with (*S*)-4-((*tert*-butoxycarbonyl)amino)-5,5-difluoropentanoic acid (1.0 g, 3.95 mmol) dissolved in 20 mL of dichloromethane (DCM). The reaction mixture was cooled to 0 ℃, and triethylamine (TEA, 1.2 equivalents, 4.74 mmol, 660 µL) was added slowly. After stirring for 10 minutes, ethyl chloroformate (ECF, 1.2 equivalents, 4.74 mmol, 451 µL) was added gradually over a 60-minute period. The reaction continued to stir at 0 ℃ for another 4 hours. Separately, in a 50 mL pear-shaped flask, 2-fluorophenyl hydrazine hydrogen chloride (1.2 equivalents, 4.74 mmol, 579 mg) and TEA (1.2 equivalents, 4.74 mmol, 660 µL) were dissolved in 10 mL of DCM. This solution was carefully introduced into the primary reaction mixture over the course of an hour, keeping the temperature at 0 ℃. After completing the hydrazine addition, the reaction was warmed to room temperature and stirred for another 16 hours. Upon completion, the organic layer was washed sequentially with distilled water, 1 N hydrochloric acid, 0.5 N sodium bicarbonate (NaHCO_3_), and then rinsed again with distilled water. The organic phase was separated, dried over anhydrous sodium sulfate (Na_2_SO_4_), and concentrated using a rotary evaporator. The target product was purified through recrystallization from a mixture of ethyl acetate and n-hexane (1:5). The yield of RA-PR058-Boc was 83%, yielding 1.18 g of the product.

Synthesis of (*S*)-4-amino-5,5-difluoro-*N*'-(2-fluorophenyl)pentanehydrazide hydrochloride (RA-PR058): A 250 mL round-bottom flask fitted with a magnetic stir bar was loaded with RA-PR058-Boc (1.15 g, 3.18 mmol). The compound was dissolved in 50 mL of 1 M hydrochloric acid (HCl) in ethyl acetate (EA, 50 mmol), and the reaction was allowed to proceed at room temperature (24 ℃) for approximately 18 hours. Afterward, the resulting white solid was filtered and washed sequentially with EA and n-hexane. The filtered solid was then dried under vacuum to yield the RA-PR058. The yield of RA-PR058 was 90%, yielding 0.85 g of the product.

### 2.3. Primary neuron culture

Hippocampal CA1-CA3 regions were isolated from postnatal (0–3 d old) 5xFAD mice. For dissociation, the dissected tissues were incubated with trypsin (Sigma) and DNase (Sigma) for 10 minutes at 37 ℃, followed by gentle trituration with pipette. 4.0–5.0 × 10^4^ cells were plated on poly-ornithine-coated coverslips. Cells were maintained in culture media consisting of Neurobasal^™^-A Medium (Gibco) supplemented with GlutaMAX^™^ Supplement (Gibco) and B-27^™^ Supplement (Gibco) in a humidified incubator set at 37 ℃ with 5% CO_2_. Neurons were transfected 7 days after plating and further incubated for 14–21 days in culture medium. All results are obtained from at least three independent primary cultures. Animal treatments in this study were carried out in accordance with Animal Care and Use Guidelines, and all experiments were approved by the Animal Care Committee of Kyung Hee University (KHSASP-24-266).

### 2.4. Optical imaging with RA-PR058 treatments

For optical imaging, primary cultured hippocampal neurons were transfected with vGlut1-pHluorin (vG-pH) using the Ca^2+^ phosphate precipitation method, as previously described (Bae et al. (2020)). Briefly, vG-pH was incubated with 2x HeBS (273 mM NaCl, 9.5 mM KCl, 1.4 mM Na_2_HPO_4_·PO_2_O, 15 mM D-glucose, 42 mM HEPES, pH 7.10) containing 2 mM Ca^2+^, after which the mixture was applied to hippocampal neurons cultured for 8 days *in vitro* (DIV8). Live-cell imaging was performed on DIV14-21 neurons transfected with vG-pH 7 days after plating. Neurons were treated with 5 μM of RA-PR058 for 6 hours. Coverslips containing neurons were mounted in a laminar-flow-perfused stimulation chamber on the stage of a custom-built, laser-illuminated epifluorescence microscope (Zeiss Observer). Live-cell images were acquired with an Andor iXon Ultra 897 (Model #DU-897U-CS0-#BV) back-illuminated EMCCD camera. A diode-pumped OBIS 488 laser (Coherent), shuttered by synchronizing the TTL on/off signal from the EMCCD camera during acquisition, was utilized as a light source. Fluorescence excitation/emission and collection were achieved using a 40 × Fluar Zeiss objective lens (1.3 NA) and 500–550 nm emission and 498 nm dichroic filters (Chroma). Action potentials (APs) were evoked by passing a 1-ms current pulse through platinum–iridium electrodes from an isolated current stimulator (World Precision Instruments). Neurons were perfused with Tyrode’s buffer consisting of 119 mM NaCl, 2.5 mM KCl, 2 mM CaCl_2_, 2 mM MgCl_2_, 25 mM HEPES, 30 mM glucose, 10 mM 6-cyano-7-nitroquinoxaline-2,3-dione, and 50 mM D,L-2-amino-5-phosphonovaleric acid, adjusted to pH 7.4. All experiments were carried out at 30 °C. All images were acquired at 2 Hz with a 50-ms exposure.

### 2.5. Image analysis

All images were analyzed using ImageJ with plugin Time Series Analyzer. Synaptic boutons were selected as oval regions of interest (diameter, 10 pixels), and the intensity of fluorescence at synapses was measured. Fluorescence traces were analyzed using Origin Pro.

### 2.6. Elevated plus maze test

The elevated plus maze (EPM) test is a widely used behavioral assay to assess anxiety-like behavior in mice (Walf and Frye 2007). After administering RA-PR058 for 1 month, EPM was performed to evaluate its effects. Briefly, the mouse was placed in the center of the maze, facing an open arm, and allowed to explore freely for 5 minutes. Behavioral patterns were recorded and analyzed by EthoVision software (Noldus). Data were used to assess the effects of RA-PR058 on anxiety-like behavior compared to control mice.

### 2.7. Cell culture and chemicals treatment

Human neuroblastoma cell line, SH-SY5Y were maintained in DMEM (Welgene) supplemented with 10% fetal bovine serum (FBS, Gibco) and 1% penicillin/streptomycin (Capricorn) at 37 ℃ in a humidified atmosphere containing 5% CO_2_. SH-SY5Y cells were treated with 10 μM of RA-PR058 for 24 hours followed by exposure to 200 μM of hydrogen peroxide (H_2_O_2_, Sigma) for 4 hours, based on previously reported methods with minor modifications (Kim et al. 2024).

### 2.8. Brain tissue preparation

After EPM was conducted, mice were sacrificed, and brain tissues were collected as previously described (Park et al. 2021). Mice were anesthetized by injecting Zoletil (Virbac) and Rompun (Bayer) intraperitoneally. After mice were anesthetized, they were perfused with phosphate-buffered saline (PBS). Then, brains were extracted and cut into two hemispheres; one hemisphere was preserved in 4% paraformaldehyde and the other was dissected into the cortex and hippocampus. Dissected tissues were then snap-frozen in liquid nitrogen and stored at −80 ℃ until further biochemical analysis.

### 2.9. Western blot analysis

Samples for western blot analysis were prepared as previously described (Gwon et al. 2012). Cells were lysed with T-PER^™^ Tissue Protein Extraction Reagent (Thermo Fisher Scientific) supplemented with protease/phosphatase inhibitor cocktail (Thermo Fisher Scientific) and incubated at 4 ℃ for 10 minutes. After incubation, lysed samples were centrifuged at 13,000 x rpm for 10 minutes at 4 ℃ and the supernatant were used for western blot analysis. The cortices and hippocampi tissues were homogenized using RIPA lysis buffer (Millipore) supplemented with protease/phosphatase inhibitor cocktail and incubated at 4 ℃ for 30 minutes. Following incubation, lysed tissues were centrifuged at 13,000 x rpm for 30 minutes at 4 ℃ and the supernatant were used for western blot analysis. Protein concentrations were measured using Pierce^™^ BCA Protein Assay Kits (Thermo Fisher Scientific). Equal amounts of proteins were mixed with NuPAGE^™^ LDS Sample Buffer (Invitrogen) and 5% 2-mercaptoethanol (Sigma) and then boiled at 95 ℃ for 5 minutes. 4–12 μg of proteins were loaded onto SDS-polyacrylamide gels and electrophoresed (SDS-PAGE) until the protein bands were fully separated. After SDS-PAGE, the protein bands were transferred onto polyvinylidene fluoride (PVDF) membranes (Millipore). Following transfer, PVDF membranes were blocked with 5% non-fat skim milk in tris-buffered saline with Tween20 (Junsei) (TBS-T) for 1 hour at room temperature. Then, PVDF membranes were incubated overnight at 4 ℃ with primary antibodies against APP (6E10, BioLegend, 803001, 1:1,000), AT8 (Invitrogen, MN1020, 1:1,000), AT180 (Invitrogen, MN1040, 1:1,000), BACE1 (Cell Signaling Technology, 5606, 1:1,000), β-Actin (Sigma, A5441, 1:10,000), p-JNK (Cell Signaling Technology, 4671, 1:1,000), JNK (Cell Signaling Technology, 9252, 1:1,000), p-Tau^S396^ (Invitrogen, 44-752G, 1:1,000), Tau13 (BioLegend, 835201, 1:1,000). Following incubation, membranes were washed with TBS-T and incubated with horseradish peroxidase-conjugated anti-mouse secondary antibody (Sigma, AP124P, 1:5,000) or anti-rabbit secondary antibody (Invitrogen, 31460, 1:5,000) for 1 hour at room temperature. Then, membranes were washed with TBS-T and visualized using Pierce^™^ ECL Western Blotting Substrate (Thermo Fisher Scientific). Protein bands were quantified using ImageJ (NIH).

### 2.10. Frozen section of brain tissue

After sacrifice, brain tissues were preserved in 4% paraformaldehyde and subsequently immersed in 10%, 20% and 30% sucrose solutions in PBS (Junsei), each for one day at 4 ℃. Tissues were then embedded in FSC 22 Frozen Section Media (Leica Biosystems) and stored at −80 ℃ until further processing. Frozen brain tissues were cryosectioned at a thickness of 30 μm using a cryostat (Leica Biosystems) and stored in a cryoprotectant solution (Biosolution) at −20 ℃.

### 2.11. DAB staining

3,3'-Diaminobenzidine (DAB) staining was performed as previously (Baek et al. 2017; Kim et al. 2024). Brain slices stored in cryoprotectant solution were floated in PBS. Using the Mouse and Rabbit Specific HRP/DAB Detection IHC kit (Abcam), brain tissues were stained for AT8. Briefly, brain tissues were treated with a hydrogen peroxide block, followed by washing with 0.3% Triton X-100 (Sigma) in PBS (PBS-T). After blocking, tissues were incubated with a protein block. Tissues were then incubated with AT8 antibody for overnight at 4 ℃. Following incubation, tissues were washed with PBS-T and treated with biotinylated goat anti-polyvalent, washed again with PBS-T, and incubated with streptavidin peroxidase. Subsequently, tissues were washed with PBS-T and treated with a mixture of DAB chromogen and DAB substrate. The color change resulting from staining was observed under a microscope. Once the desired staining intensity was reached, tissues were washed with PBS-T and mounted on the slides.

### 2.12. RNA sequencing analysis

Total RNA was extracted from the cortex of mice using RNAiso Plus (Takara Bio) according to the manufacturer’s instructions and used for mRNA sequencing analysis. RNA purity and concentration were measured using Take 3 (BioTek). Integrity of RNA was determined using 2100 Bioanalyzer Instrument (Agilent Technologies). mRNA library preparation and transcriptomic analysis with data quality control was carried out by Novogene Co., LTD (Hong Kong). For the data quality control, fastq files for all RNA sequencing samples were filtered and adapters were trimmed. Following trimming, the reads were aligned to the mouse reference genome GRCm38 by the STAR Aligner. Genes with fold change ≥ 1.3 and *p*-value < 0.05 were regarded as differentially expressed.

### 2.13. Microsomal stability

The metabolic stability of RA-PR058 was evaluated using liver microsomes from humans and various animal species at 37 ℃. This assay was carried out by SPMED Co., Ltd (Republic of Korea). The test was initiated by adding the 1 μM of RA-PR058 to liver microsomes in the presence or absence of an NADPH regenerating system. After incubation at 37 ℃ for 30 minutes, acetonitrile containing an internal standard was added to each sample to terminate the reaction. Samples were then centrifuged, and the supernatant was analyzed by LC-MS.

### 2.14. Cytochrome P450 inhibition assay

Inhibitory potentials of RA-PR058 on cytochrome P450 (CYP) isoforms (CYP1A2, CYP2C9, CYP2C19, CYP2D6, and CYP3A) were evaluated. This assay was conducted by SPMED Co., Ltd (Republic of Korea). Human liver microsomes were incubated at 37 ℃ for 20 minutes with phosphate buffer (pH 7.4), each CYP substrate (for CYP1A2: phenacetin, CYP2C9: diclofenac, CYP2C19: S-mephenytoin, CYP2D6: dextromethorphan, CYP3A: midazolam), and 10 μM of RA-PR058 in the presence of an NADPH regenerating system. The reaction was terminated by adding an acetonitrile solution containing an internal standard. Following reaction termination, samples were centrifuged, and the supernatant was injected into an LC-MS/MS system to analyze the metabolites of each substrate drug.

### 2.15. Pharmacokinetic measurements

Pharmacokinetic assays were conducted by NeuroVis (Republic of Korea). 10 mg/kg of RA-PR058 was administered to Sprague–Dawley rats by either oral administration route or intravenous injection route. Blood sampling was conducted using the BASi Culex ABS system. After cannulation, catheters were inserted into the jugular vein and the carotid artery of the rats, with the system programmed to collect 200 μL samples. Collected blood samples were centrifuged at 12,000 x rpm for 10 minutes to separate plasma, which was then immediately stored at −80 ℃. For brain pharmacokinetics, 10 mg/kg of RA-PR058 was administered orally to Sprague–Dawley rats, followed by collection of both blood and brain tissue. After collection, samples were mixed with the internal standard, ofloxacin, and centrifuged at 13,000 x rpm for 5 minutes at 4 ℃. The resulting supernatants were then combined with 50% methanol and analyzed by LC-MS/MS.

### 2.16. Statistics

Graphs were created and statistical analysis were carried out using Prism 8 software (GraphPad Software) or Origin Pro (OriginLab). Data were analyzed using unpaired two-tailed t-test or one-way ANOVA with Dunnett’s multiple comparisons test. All data are presented as mean ± standard deviation (SD) or standard error of the mean (SEM).

## 3. Results

### 3.1. RA-PR058 reduces BACE1 expression *in vitro*

**Figure 1.**
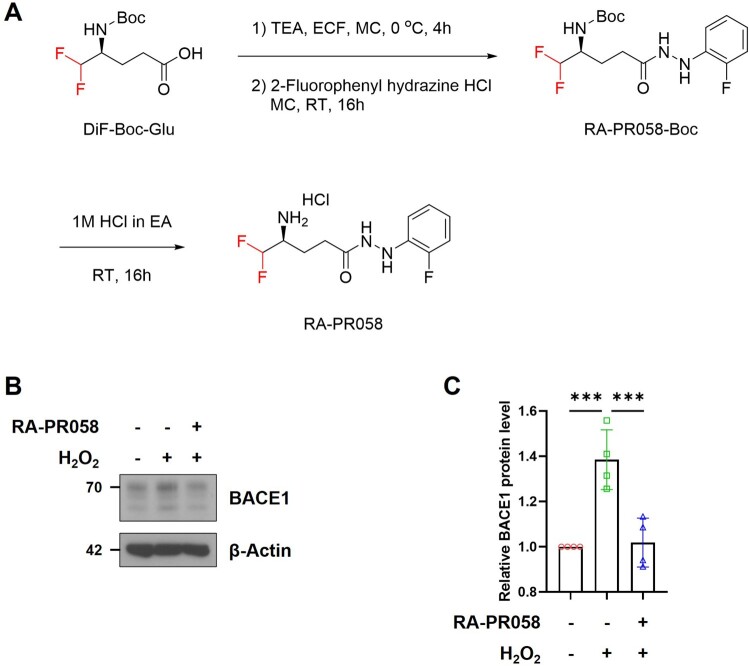
Synthesis of RA-PR058 and its regulatory effects on BACE1 expression. (A) Synthesis scheme of RA-PR058. (B) Representative western blot image of BACE1 in SH-SY5Y. SH-SY5Y cells were incubated with 10 μM of RA-PR058 for 24 hours followed by exposure to 200 μM of H_2_O_2_ for 4 hours. (C) Quantitative analysis of BACE1 protein expression levels in (B). BACE1 protein expression levels were normalized with β-Actin (*n *= 4). Data are shown as mean ± SD in (C). EA: Ethyl acetate, ECF: Ethyl chloroformate, TEA: Triethylamine. Statistical significance was assessed by one-way ANOVA with Dunnett’s multiple comparisons. ***p < 0.001.

To enhance the therapeutic efficacy of ramalin, we designed and synthesized a novel ramalin derivative, RA-PR058 (Figure 1A). To examine the therapeutic effects of RA-PR058 on AD-related pathologies, we utilized *in vitro* systems with SH-SY5Y neuronal cell line. We investigated whether RA-PR058 regulates BACE1 expression under oxidative stress conditions in SH-SY5Y cells. Upon treatment with hydrogen peroxide (H₂O₂), BACE1 expression was significantly upregulated, however, RA-PR058 treatment markedly attenuated this H₂O₂-induced increase in BACE1 expression (Figure 1B, C) (Mouton-Liger et al. 2012; Jo et al. 2010).

### 3.2. RA-PR058 partially restores synaptic transmission function

**Figure 2.**
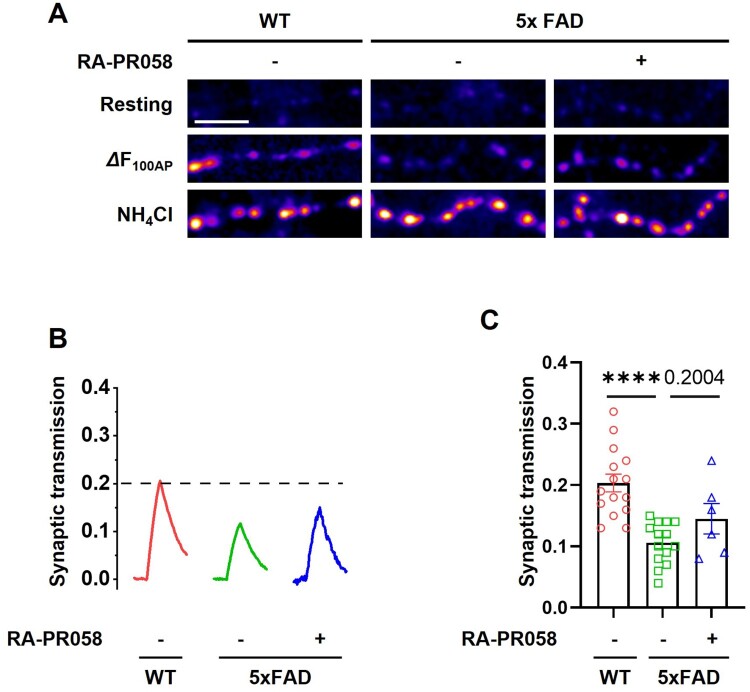
RA-PR058 restores synaptic function in 5xFAD hippocampal neurons. (A) Representative images of vG-pH at resting (top), 100 AP (middle), and NH_4_Cl (bottom) neurons (Scale bar, 5 mm). (B) Representative ensemble average traces of vG-pH in response to 100AP in WT (red), 5xFAD (green), and RA-PR058 treated 5xFAD (blue) hippocampal neurons. Neurons expressing vG-pH were stimulated at 10 Hz for 10s in the presence or absence of RA-PR058. Intensities were normalized to the maximal value of NH_4_Cl response. (C) Mean values of amplitudes of 100AP responses in WT, 5xFAD, and RA-PR058 treated 5xFAD hippocampal neurons. [WT] = 0.20 ± 0.01 (*n *= 15); [5xFAD] = 0.11 ± 0.01 (*n *= 14); [RA-PR058 treated 5xFAD] = 0.15 ± 0.03 (*n *= 6). Data are shown as mean ± SEM in (C). Statistical significance was assessed by one-way ANOVA with Dunnett’s multiple comparisons. ****p < 0.0001.

Synaptic dysfunction is a critical aspect of AD pathology (Shankar and Walsh 2009). To evaluate the potential of RA-PR058 to restore synaptic transmission, we conducted a pHluorin-based assay with pHluorin conjugated to the luminal region of synaptic vesicle membrane proteins such as vesicular glutamate transporter 1 (vGlut1). 5xFAD mouse model, which harbors human APP and presenilin 1 (PS1) transgenes with five AD-linked mutations, is widely used to investigate AD-related pathologies such as Aβ accumulation, synaptic dysfunction and cognitive deficit. Hippocampal neurons derived from wild-type (WT) and 5xFAD mice were incubated with 10 μM of RA-PR058 and subsequently stimulated with 100 action potentials (AP) at 10 Hz. Fluorescence intensity was measured to quantify synaptic transmission (Figure 2A). Neurons derived from 5xFAD hippocampus exhibited significant impairment in synaptic transmission compared to those from WT hippocampus neurons. Although the RA-PR058 treatment partially recovered this synaptic transmission disfunction, this recovery did not reach statistical significance (Figure 2B, C). Nevertheless, the observed trend suggests a potential effect of RA-PR058 in restoring synaptic transmission function impaired in the context of AD.

### 3.3. RA-PR058 reduces anxiety-like behavior, BACE1 expression, and tau phosphorylation in 3xTg-AD mice

**Figure 3.**
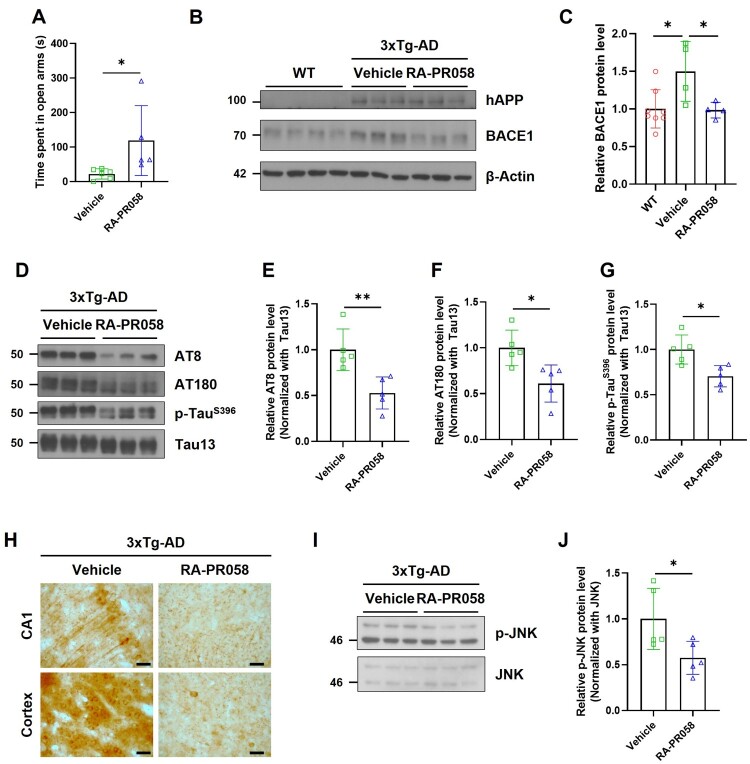
RA-PR058 alleviates anxiety-like behavior and reduces BACE1 expression and phosphorylated tau levels in the brains of 3xTg-AD mice. Seven-month-old 3xTg-AD mice were orally administered with 20 mg/kg of RA-PR058 daily for 4 weeks, followed by the EPM test and biochemical analysis of brain tissues. (A) Time spent in open arms of the EPM by vehicle-treated, and RA-PR058-treated 3xTg-AD (*n *= 5-6 per group). (B) Representative western blot images of hAPP and BACE1 in the cortex. (C) Quantitative analysis of BACE1 protein expression levels in (B). BACE1 protein expression levels were normalized with β-Actin. (*n *= 4-8 per group). (D) Representative western blot images of AT8, AT180, p-Tau^S396^ and Tau13 in the cortex. (E-G) Quantification of AT8 protein expression levels (E), AT180 protein expression levels (F), and p-Tau^S396^ protein expression levels (G) in (D). AT8, AT180, and p-Tau^S396^ protein expression levels were normalized with Tau13. (H) Immunohistochemistry analysis of AT8 in the brains of 3xTg-AD group (Scale bar, 50 μm). (I) Representative western blot images of p-JNK and JNK in the cortex. (J) Quantitative analysis of p-JNK protein expression levels in (I). p-JNK protein expression levels were normalized with JNK. Data are shown as mean ± SD in (A), (C), (E), (F), (G), and (J). Statistical significance was assessed by unpaired two-tailed t-test for (A), (E), (F), (G), and (J) or one-way ANOVA with Dunnett’s multiple comparisons for (C). *p < 0.05; **p < 0.01.

3xTg-AD mouse model, which contains three mutations related to familial AD (APP Swedish, MAPT P301L, and PSEN1 M146 V) is extensively utilized to investigate AD pathologies, including Aβ accumulation and tau pathologies. Seven-month-old 3xTg-AD mice were treated with RA-PR058 for 1 month, after which behavioral and biochemical analyses were conducted. In the elevated plus maze (EPM) test, RA-PR058 treated 3xTg-AD group spent significantly more time in the open arms compared to the vehicle-treated 3xTg-AD group, suggesting that RA-PR058 alleviated anxiety-like behaviors in the 3xTg-AD mice (Figure 3A).

To assess the effect of RA-PR058 on AD-related molecular factors, we examined BACE1 expression and tau phosphorylation in the cortex of 3xTg-AD mice. BACE1 expression was elevated in the vehicle-treated 3xTg-AD group compared to WT controls. However, RA-PR058 treatment significantly reduced cortical BACE1 expression (Figure 3B, C). Additionally, RA-PR058 treatment significantly reduced tau phosphorylation at multiple sites associated with AD pathology, including Ser202/Thr205 (AT8), Thr231 (AT180), and Ser396 in the cortex (Figure 3D–H). Moreover, treatment with RA-PR058 significantly downregulated JNK phosphorylation, implying that RA-PR058 regulates tau phosphorylation by mediating JNK phosphorylation (Figure 3I, J).

In the hippocampus, however, RA-PR058 exhibited region-specific effects. While RA-PR058 treatment significantly reduced AT8 expression, it did not affect BACE1 expression or other phosphorylated tau species (Supplementary Figure S1).

These findings indicate that RA-PR058 not only alleviates anxiety-like behavior but also modulates key molecular factors of AD-related pathology in 3xTg-AD mice.

### 3.4. Transcriptomic analysis reveals RA-PR058-mediated gene expression changes in 3xTg-AD mice

**Figure 4.**
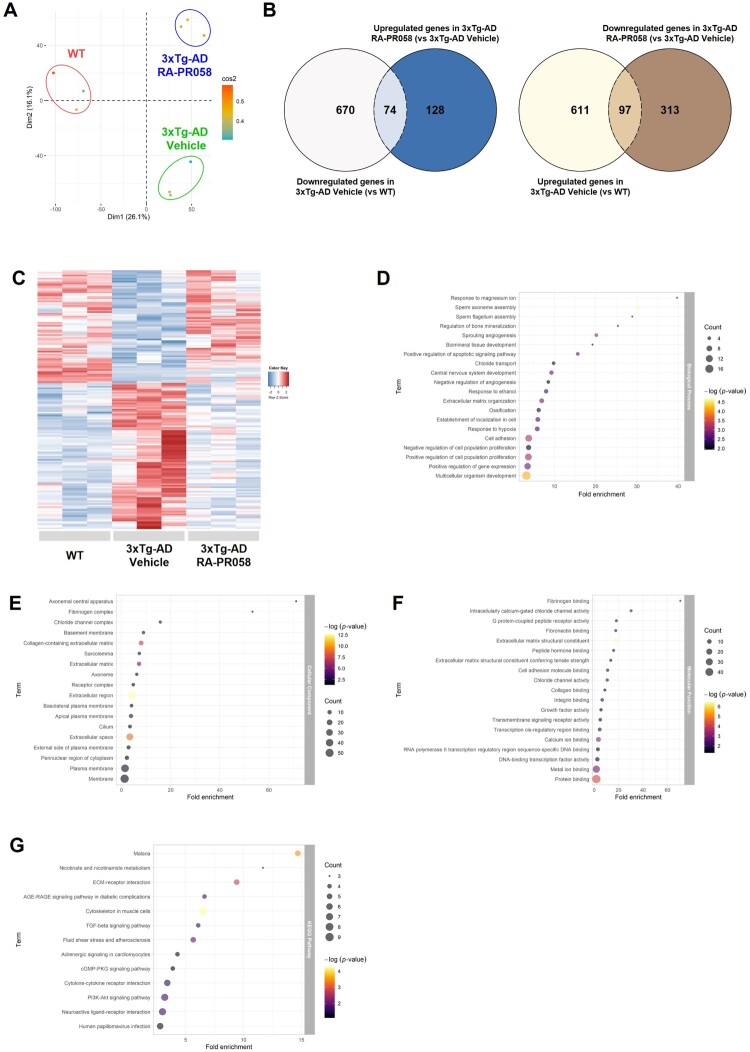
Transcriptomic analysis of gene expression changes induced by RA-PR058 treatment in 3xTg-AD mice. Total RNA from cortices was used for RNA-sequencing analysis (*n *= 3 per group). Differentially expressed genes (DEGs) were defined as those with a fold change ≥ 1.3 and *p*-value < 0.05. (A) Principal component analysis (PCA) of samples from WT, 3xTg-AD Vehicle, and 3xTg-AD RA-PR058 groups. (B) Venn diagram demonstrating the overlap of DEGs between groups. (C) Heatmap showing expression profiles of 171 DEGs identified in (B). (D–F) Gene ontology (GO) enrichment analysis of the 171 DEGs. (D) Top 20 enriched GO terms in the biological process category. (E) Top 18 enriched GO terms in the cellular component category. (F) Top 19 enriched GO terms in the molecular function category. (G) Top 13 enriched KEGG pathway terms based on analysis of the 171 DEGs.

To explore the genomic impact of RA-PR058 treatment, we performed RNA-sequencing analysis on cortical RNA from 3xTg-AD and WT mice. Principal component analysis (PCA) showed clear clustering between the treatment groups indicating distinct gene expression profiles (Figure 4A). Differentially expressed genes (DEGs) analysis identified 1,452 DEGs between the 3xTg-AD vehicle group and the WT group, and 612 DEGs between the RA-PR058-treated 3xTg-AD group and the vehicle-treated 3xTg-AD group (Figure 4B).

Among these DEGs, 74 genes were shared between genes downregulated in the 3xTg-AD vehicle group (compared to WT) and genes upregulated in the RA-PR058-treated 3xTg-AD group (compared to the 3xTg-AD vehicle group). Similarly, 97 genes were shared between upregulated genes in the 3xTg-AD vehicle group (vs. WT) and genes downregulated in the RA-PR058-treated 3xTg-AD group (vs. 3xTg-AD Vehicle) (Figure 4B, C).

We conducted gene ontology (GO) analysis and Kyoto Encyclopedia of Genes and Genomes (KEGG) pathway analysis on these 171 overlapping DEGs (Figure 4D–G). In the GO biological process category, genes involved in ‘Central nervous system development’, ‘Negative regulation of cell population proliferation’, and ‘Response to hypoxia’ were significantly enriched (Figure 4D). In the cellular component category, genes associated with ‘Axonemal central apparatus’, ‘Axoneme’, and ‘Cilium’ were significantly enriched (Figure 4E). Additionally, in the molecular function category, genes related to ‘Fibrinogen binding’, ‘Extracellular matrix structural constituent’, and ‘Calcium ion binding’ were significantly enriched (Figure 4F). KEGG pathway analysis highlighted significant enrichment in pathways related to the ‘AGE-RAGE signaling pathway in diabetic complications’, ‘TGF-beta signaling pathway’, ‘Cytokine-cytokine receptor interaction’, and ‘Neuroactive ligand–receptor interaction’ (Figure 4G, Supplementary Figure S2). To obtain more detailed insights, we focused on specific genes within these pathways that showed significant expression changes. For instance, in the ‘AGE-RAGE signaling pathway in diabetic complications’, vascular cell adhesion molecule 1 (Vcam1) was significantly increased in the 3xTg-AD vehicle group compared to the WT group and was reduced by RA-PR058 treatment (Supplementary Figure S2A). Similarly, bone morphogenic protein 6 (Bmp6), which is part of both the ‘TGF-beta signaling pathway’ and ‘cytokine-cytokine receptor interaction’, was elevated in the 3xTg-AD vehicle group compared to the WT group and showed a reduction after RA-PR058 treatment (Supplementary Figure S2B, C).

### 3.5. Pharmacokinetics of RA-PR058

**Figure 5.**
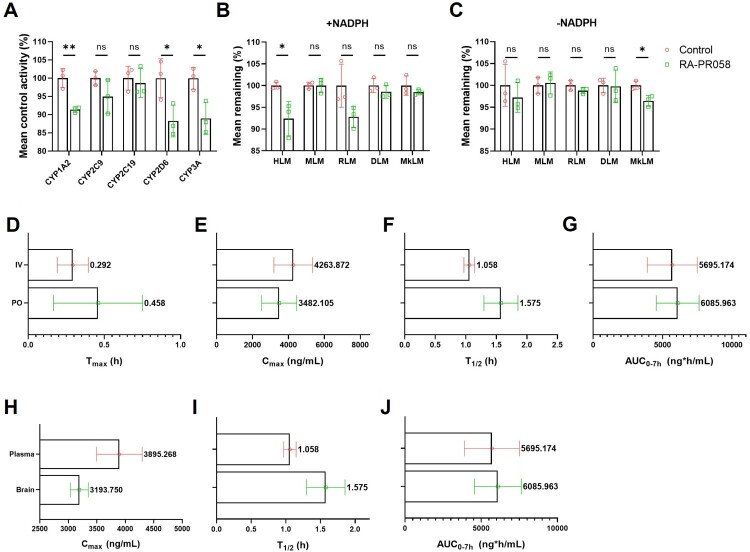
Pharmacokinetics of RA-PR058. (A) Potential of inhibitory interaction of RA-PR058 on cytochrome P450 isoform activities (*n *= 3 per group). (B, C) Metabolic stability of RA-PR058 in liver microsomes from human (HLM), mouse (MLM), rat (RLM), dog (DLM), and monkey (MkLM) with (B) and without (C) NADPH (*n *= 3 per group). (D-G) Pharmacokinetic parameters of RA-PR058 following intravenous (IV) injection and oral (PO) administration in S.D. rats (*n *= 3 per group). (H-J) Pharmacokinetic parameters of RA-PR058 in plasma and brain tissues of S.D. rats (*n *= 3 per group). Data are shown as mean ± SD. Mean value is presented alongside error bars on the graphs in (D-J). Statistical significance was assessed by unpaired two-tailed t-test for (A), (B), and (C). Non-significant (ns) *p *> 0.05; *p < 0.05; **p < 0.01.

We assessed the pharmacokinetic parameters of RA-PR058, including its inhibitory interaction potential with cytochrome P450 (CYP) isoforms, metabolic stability, and blood–brain barrier permeability. To evaluate CYP inhibition, we measured the effect of RA-PR058 on CYP isoform activity and observed a 12% decrease in mean control activity, indicating minimal interaction with CYP enzymes (Figure 5A) (Lin et al. 2007).

Additionally, metabolic stability was assessed using liver microsomes, where the mean remaining (%) of RA-PR058 concentration was greater than 90% across all microsomes tested, suggesting that RA-PR058 is a highly stable compound (Figure 5B, C).

We also examined the pharmacokinetic parameters of RA-PR058 following intravenous (IV) and oral (PO) administration in Sprague–Dawley rats (Figure 5D–G). After oral administration, we measured the area under the curve (AUC) in plasma and brain tissue. The brain-to-plasma AUC ratio (AUC₀–₇_h_) was 0.916, indicating that RA-PR058 can cross the blood–brain barrier to a limited extent (Figure 5H–J).

These findings suggest that RA-PR058 is a promising candidate for AD treatment, combining low CYP interaction risk, high metabolic stability, and the potential for CNS accessibility.

## 4. Discussion

AD is a complex, multifactorial disorder driven by various underlying mechanisms rather than a single cause. For several decades, Aβ has been regarded as a primary therapeutic target in AD research. As a key enzyme in the amyloidogenic pathway of APP processing, BACE1 activity and expression are significantly upregulated in AD brains compared to non-demented controls, making BACE1 a focal point in AD treatment strategies (Yang et al. 2003). However, despite extensive efforts to develop BACE1 inhibitors, most clinical trials have failed due to severe side effects, likely stemming from off-target effects on the physiological substrates of BACE1. Therefore, modulating BACE1 expression, rather than directly inhibiting its enzymatic activity, may provide a safer and more effective therapeutic approach for AD. Supporting this concept, recent studies have shown that downregulation of BACE1 expression through epigenome editing in AD animal models can attenuate cognitive deficits and reduce Aβ plaques accumulation (Han et al. 2024).

Natural products have been widely investigated for their therapeutic potential in treating various diseases, including wound healing, obesity, inflammation, and AD (Kim et al. 2022; Mun et al. 2024; Kim et al. 2024; Shin et al. 2024). Among these, ramalin, a bioactive compound derived from the Antarctic lichen, *Ramalina terebrata*, has garnered attention for its potent antioxidant, anticancer, antibacterial, and anti-inflammatory properties (Suh et al. 2017; Paudel et al. 2011; Paudel et al. 2010; Kim et al. 2021). Building upon these findings, our study explored the effects of the novel ramalin derivative, RA-PR058 on AD pathology. We demonstrated that RA-PR058 effectively reduced oxidative stress-induced BACE1 expression *in vitro* and decreased cortical BACE1 expression in 3xTg-AD mice.

**Figure 6.**
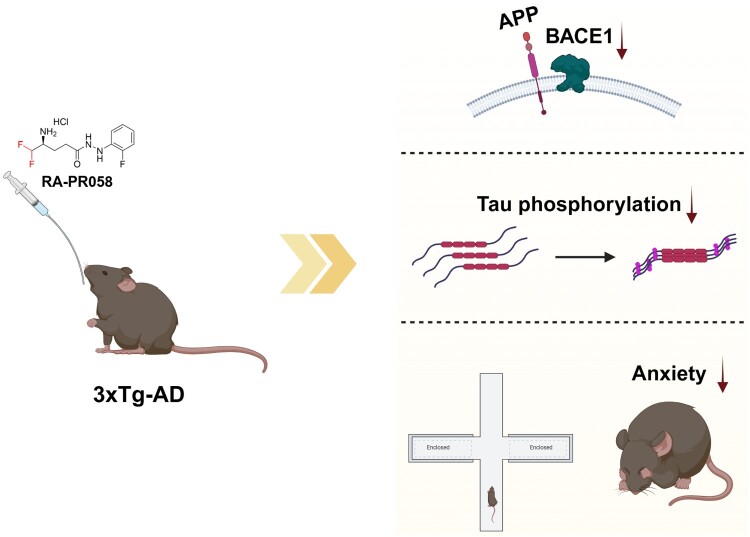
Illustration of the effects of RA-PR058 on 3xTg-AD mouse models. Administration of RA-PR058 into 3xTg-AD reduces BACE1 expression and tau phosphorylation as well as alleviating anxiety-like symptoms. Illustration is created with Biorender.com

In addition to BACE1 modulation, we confirmed the therapeutic effects of RA-PR058 *in vivo* using 3xTg-AD mice. One month of oral administration of RA-PR058 significantly alleviated anxiety-like behavior, decreased BACE1 expression, and reduced tau phosphorylation at AD-related sites, including Ser202/Thr205 (AT8), Thr231 (AT180), and Ser396. These findings underline the therapeutic potential of RA-PR058 as a therapeutic agent that targets multiple AD-related pathologies (Figure 6).

Our transcriptomic analysis of cortical tissues from RA-PR058-treated 3xTg-AD mice identified several DEGs when compared to WT and vehicle-treated 3xTg-AD groups. Of these DEGs, 171 DEGs exhibited expression patterns indicative of RA-PR058-mediated modulation: genes downregulated in the 3xTg-AD vehicle-treated group were upregulated in the RA-PR058-treated group, and vice versa. GO and KEGG pathway analyses of these DEGs revealed significant enrichment in pathways related to ‘Central nervous system development’, ‘Response to hypoxia’, ‘Calcium ion binding’, ‘Cytokine-cytokine receptor interaction’, and ‘Neuroactive ligand–receptor interaction’.

Pharmacokinetic analysis showed that RA-PR058 has a favorable profile for potential AD treatment, with low CYP interaction, high metabolic stability, and limited but measurable brain penetration. These characteristics suggest RA-PR058 could deliver sustained therapeutic effects with minimal risk of drug–drug interactions, a critical factor in polypharmacy scenarios common in AD management.

While RA-PR058 demonstrated promising effects, including reductions in BACE1 expression and tau phosphorylation, its impact on cognitive restoration was not evident in this study. Moreover, the precise mechanisms by which RA-PR058 modulates these pathological markers remain to be elucidated. Further research is needed to investigate the molecular pathways involved and to assess the long-term cognitive benefits of RA-PR058, particularly in models with more advanced cognitive impairment.

Despite these limitations, RA-PR058 shows promise as a multi-targeted therapeutic candidate for AD treatment. By modulating key molecular markers associated with AD pathology and demonstrating a favorable pharmacokinetic profile, RA-PR058 represents a potential advancement in the development of effective, well-tolerated AD therapies.

## Supplementary Material

Supplementary Material

## Data Availability

The data used in this study were available from the corresponding author upon reasonable request.
