# Supplementary material for: RA-PR058, a novel ramalin derivative, reduces BACE1 expression and phosphorylation of tau in Alzheimer’s disease mouse models
Source: Anim Cells Syst (Seoul). 2025 Feb 7;29(1):122–34. doi: 10.1080/19768354.2025.2459649 (PMC11809180; doi:10.1080/19768354.2025.2459649)
Supplement: Supplementary Material [file TACS_A_2459649_SM2363.docx]

**RA-PR058, a novel ramalin derivative, reduces BACE1 expression, and phosphorylation of tau in Alzheimer’s disease mouse models**

**Supplementary Information**

**Supplementary Figure S1, S2**

**Supplementary Figure S1**

**
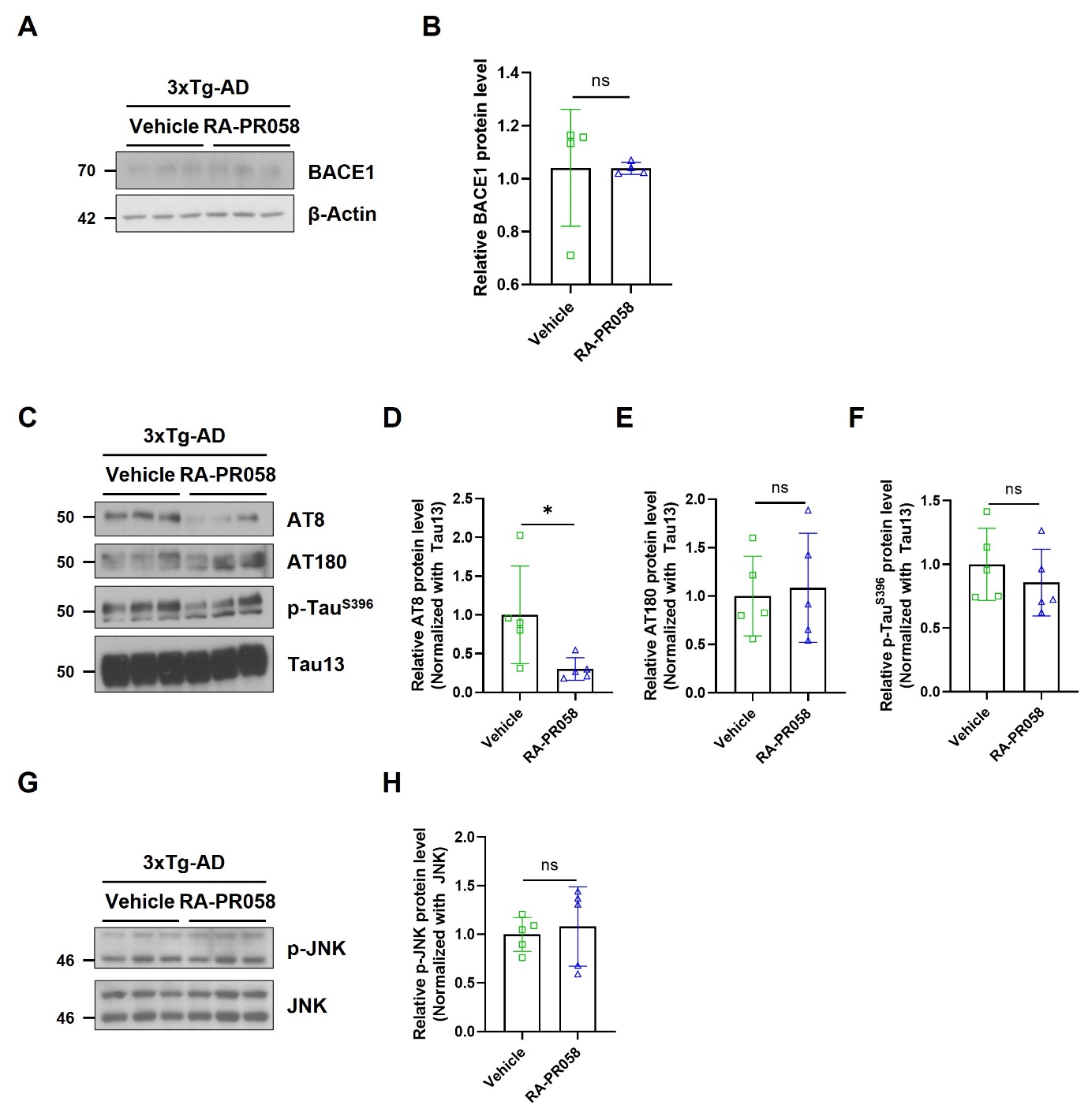
**

**Supplementary Figure S1. RA-PR058 decreases AT8 levels, not BACE1 and other phosphorylated tau species in the hippocampus of 3xTg-AD mice.** (A) Representative western blot images of BACE1 in the hippocampus. (B) Quantitative analysis of BACE1 protein expression levels in (A). BACE1 protein expression levels were normalized with β-Actin. (C) Representative western blot images of AT8, AT180, p-Tau^S396^ and Tau13 in the hippocampus. (D-F) Quantitative analysis of AT8 protein expression levels (D), AT180 protein expression levels (E), and p-Tau^S396^ protein expression levels (F) in (C). AT8, AT180, and p-Tau^S396^ protein expression levels were normalized with Tau13. (G) Representative western blot images of p-JNK and JNK in the hippocampus. (H) Quantitative analysis of p-JNK protein expression levels in (G). p-JNK protein expression levels were normalized with JNK. Data are shown as mean ± SD in (B), (D), (E), (F), and (H). Statistical significance was assessed by unpaired two-tailed t-test for (B), (D), (E), (F), and (H). Non-significant (ns) *p* > 0.05; **p* < 0.05.


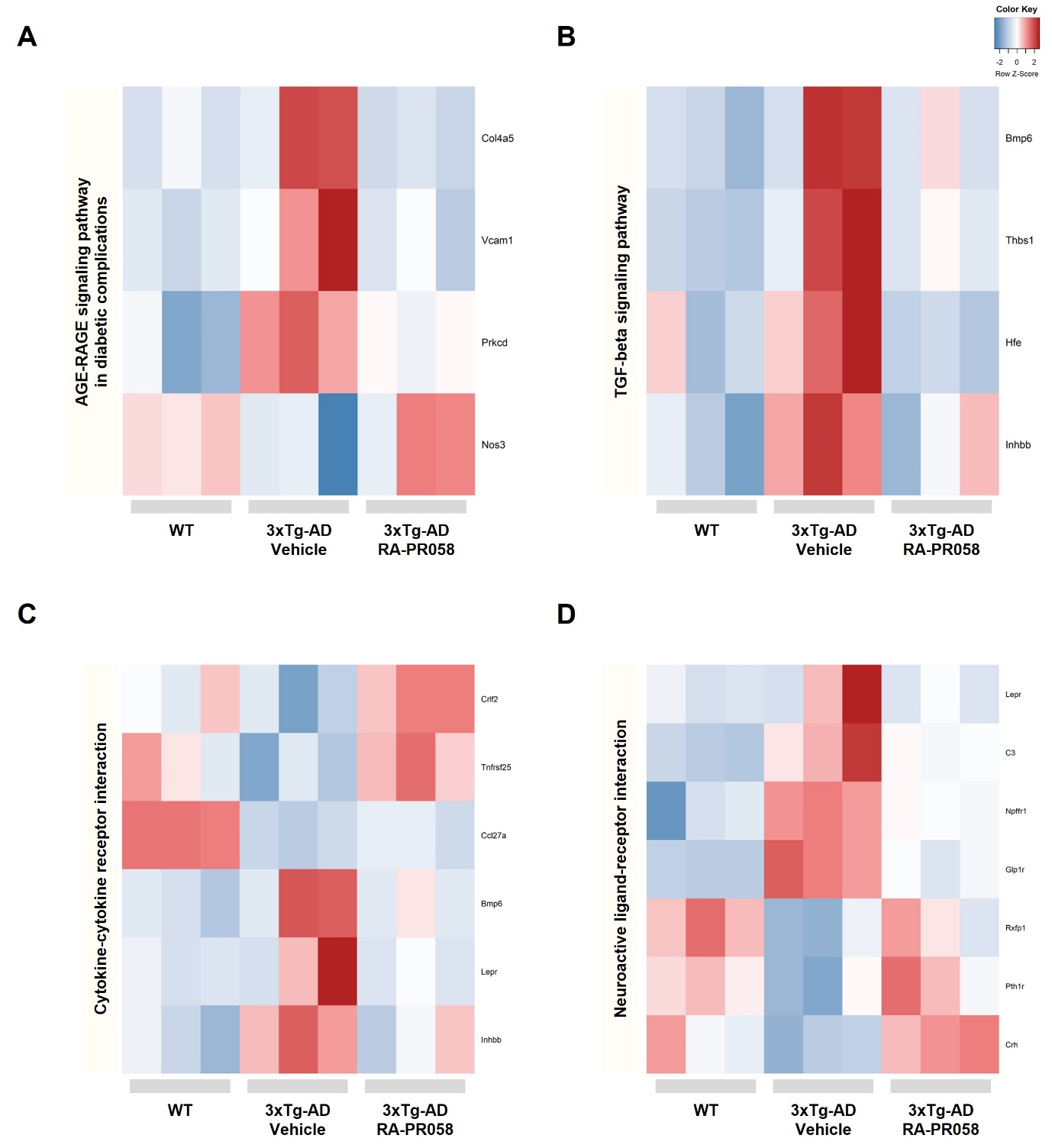


**Supplementary Figure S2. Heatmap panels of KEGG pathway affected by RA-PR058 treatment in 3xTg-AD cortex.**

Heatmap representations of (A) AGE-RAGE signaling pathway in diabetic complications-related genes, (B) TGF-beta signaling pathway-related genes, (C) Cytokine-cytokine receptor interaction-related genes, and (D) Neuroactive ligand-receptor interaction-related genes.
